# FRA1 promotes squamous cell carcinoma growth and metastasis through distinct AKT and c-Jun dependent mechanisms

**DOI:** 10.18632/oncotarget.9110

**Published:** 2016-04-29

**Authors:** Xiaoling Zhang, Joseph Wu, Suju Luo, Terry Lechler, Jennifer Y. Zhang

**Affiliations:** ^1^ Department of Dermatology, Duke University School of Medicine, Durham, NC, USA; ^2^ Department of Cell Biology, Duke University School of Medicine, Durham, NC, USA; ^3^ Department of Pathology, Duke University School of Medicine, Durham, NC, USA

**Keywords:** FRA1, AKT, c-Jun, cyclinB1, SCC

## Abstract

FRA1 (Fos-like antigen 1) is highly expressed in many epithelial cancers including squamous cell carcinoma of the skin (cSCC) and head and neck (HNSCC). However, the functional importance and the mechanisms mediating FRA1 function in these cancers are not fully understood. Here, we demonstrate that FRA1 gene silencing in HNSCC and cSCC cells resulted in two consequences – impaired cell proliferation and migration. FRA1 regulation of cell growth was distinct from that of c-Jun, a prominent Jun group AP-1 factor. While c-Jun was required for the expression of the G1/S phase cell cycle promoter CDK4, FRA1 was essential for AKT activation and AKT-dependent expression of CyclinB1, a molecule required for G2-M progression. Exogenous expression of a constitutively active form of AKT rescued cancer cell growth defect caused by FRA1-loss. Additionally, FRA1 knockdown markedly slowed cell adhesion and migration, and conversely expression of an active FRA1 mutant (FRA1DD) expedited these processes in a JNK/c-Jun-dependent manner. Through protein and ChIP-PCR analyses, we identified KIND1, a cytoskeletal regulator of the cell adhesion molecule β1-integrin, as a novel FRA1 transcriptional target. Restoring KIND1 expression rescued migratory defects induced by FRA1 loss. In agreement with these *in vitro* data, HNSCC cells with FRA1 loss displayed markedly reduced rates of subcutaneous tumor growth and pulmonary metastasis. Together, these results indicate that FRA1 promotes cancer growth through AKT, and enhances cancer cell migration through JNK/c-Jun, pinpointing FRA1 as a key integrator of JNK and AKT signaling pathways and a potential therapeutic target for cSCC and HNSCC.

## INTRODUCTION

Squamous-cell carcinoma (SCC) is a cancer of squamous epithelial cells that typically cover the surface of various tissues including skin and the oral, paranasal and nasal cavities, as well as pharynx and larynx of the head and neck. Approximately 700,000 new cases of cutaneous SCC (cSCC) and 60,000 new cases of head neck SCC (HNSCC) are estimated each year in the US, with each accounting for more than 8,000 death annually (http://www.cancer.org/acs; http://www.skincancer.org). Early stage cSCC can be effectively treated with surgical removal, whereas early stage HNSCC may require radiation due to anatomical reasons, making HNSCC one of the most difficult to treat cancers [1]. Advanced stage cancers require combination therapies involving surgery, radiation and chemotherapies such as cisplatin, a DNA alkylating agent, as well cetuximab, a monoclonal antibody against EGFR [2, 3]. These combination treatments have yielded over 50% 5-year relative survival for HNSCC [4, 5]. Despite improved outcomes with current treatments, disease recurrence and treatment toxicity, as well as excess mortality from secondary cancers continue to pose major challenges [6–8]. In order to develop novel therapeutic strategies, it is imperative to gain a better understanding of the molecular mechanisms promoting tumor growth and progression.

A plethora of oncogenes and tumor suppressors have been linked to cSCC and HNSCC [9–14]. The PI3K/AKT signaling pathway represents by far the most commonly activated pathways in HNSCC [15, 16], which is in part attributed to frequent genetic mutations affecting various components of this pathway including PIK3CA, PIK3R1, AKT and PTEN [15]. Additionally, the PI3K/AKT pathway is activated in response to signals from growth factors such as EGF and its receptor EGFR which is commonly activated in cSCC and HNSCC [17]. It is well-known that activated AKT acts through an array of transcription factors including AP-1 family gene regulators to promote cell growth and migration [18]. On the other hand, nearly 70% of HNSCC samples have no known genetic mutations directly affecting the core components of the PI3K/AKT pathway [15], suggesting that there are indirect mechanisms contributing to the activation of this pathway.

FRA1 (Fos-like antigen-1), along with c-Fos, FosB, FosB2 and FRA2, constitutes the Fos-group AP-1 transcription factors which generally function through dimerization with the Jun-group AP-1 proteins such as c-Jun, JunB and JunD. The Jun/Fos dimers regulate expression of a wide range of target genes through interaction with the consensus cis-regulatory DNA segments such as the TPA-responsive element (tga(g/c)tca) [19, 20]. As such, AP-1 plays critical roles in a wide variety of biological processes including cell growth, apoptosis, differentiation and metabolism, and is characterized as a double-edged sword in tumorigenesis and inflammation [21–23]. Different AP-1 subunits display functional diversity in a cell-type specific manner. Germline deletion of *Fra1* leads to mouse embryonic lethality due to extraembryonic tissue defects [24]. In contrast, restricting *Fra1* deletion in the embryo but not in placenta produces animals with normal growth albeit with development of osteoporosis [25]. These findings indicate that FRA1 is not required for organogenesis other than bone matrix formation.

Like other AP-1 subunits, FRA1 has been recently linked to multiple cancers, including breast, bladder, colon and esophagus cancers and HNSCC [22, 26–30]. Nevertheless, little is known about the role of FRA1 and the mechanisms mediating its function in HNSCC. Recently, it has been shown that FRA1 acts outside the nucleus to regulate membrane lipid synthesis in an AP-1-independent manner [31, 32]. In this study, we demonstrate that gene silencing of FRA1 impaired growth and migration of multiple HNSCC cell lines. Conversely, overexpression of FRA1DD, a constitutively active phosphomimetic FRA1 mutant [28], markedly enhanced cell migration. At a molecular level, loss of FRA1 inhibited AKT activation and AKT-dependent and c-Jun-independent CyclinB1 expression. In addition, FRA1 partnered with c-Jun to regulate KIND1, a cytoskeletal protein involved in β1-integrin signaling and focal adhesions. In agreement with the *in vitro* data, FRA1 loss markedly slowed subcutaneous tumor growth, and prevented metastasis *in vivo*. Our findings reveal a key role of FRA1 in AKT-activation and promotion of HNSCC cell growth, migration and metastasis.

## RESULTS

### FRA1 is required for HNSCC cell growth and resistance to chemotherapy

**Figure 1:**
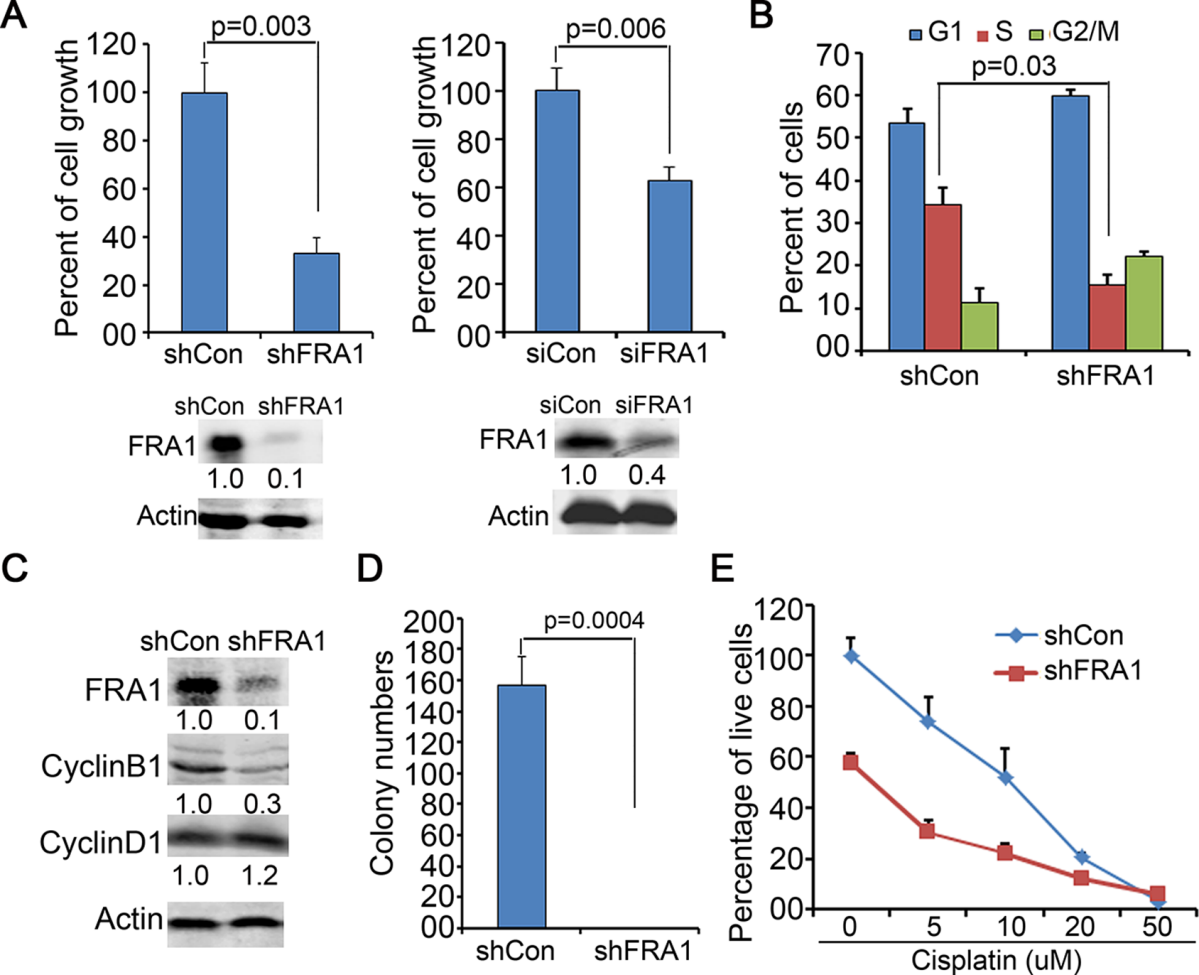
FRA1 is required for HNSCC cell proliferation (**A**) Cell growth analysis. FaDu cells were transduced in triplicates with lentivirus encoding shRNA targeting FRA1 (shFRA1) or nonsilencing control (shCon), or transfected with siRNA oligonucleotides targeting FRA1 (siFRA1) or non-silencing control (siCon). Graph represents average percentage of cell numbers normalized to that of control cells + SD at 72 h after seeding. Gene silencing was confirmed by immunoblotting as shown below each graph. (**B**) Cell cycle analysis by flow cytometry. Graph represents percentage of cells in G0/G1, S and G2/M phases + SD. (**C**) Immunoblotting for CyclinB1, CyclinD1 and Actin with protein lysates isolated at 48 h after gene transduction. (**D**) Soft-agar colony formation. Graph represents average number of colonies of triplicate dishes + SD. (**E**) Cell growth response to cisplatin. shCon or shFRA1 FaDu cells were treated in hexad with varying doses of cisplatin for 72 h, and then analyzed by MTT assay. Graph represents percentage of live cells normalized to that of untreated cells + SD. *P*-values were obtained via two tiered Student *T*-Test. Relative densitometry of immunoblots noted below each band was obtained by using Odyssey image tools.

To determine whether FRA1 is required for HNSCC cell growth, we performed gene silencing in FaDu cells via gene transduction with lentiviral shRNA (shFRA1) or transfection with siRNA oligonucleotides (siFRA1). Cell growth analysis revealed that FRA1 gene silencing by both approaches, as confirmed by immunoblotting, markedly reduced cell growth (Figure 1A). Flow cytometry of BrdU labelled cells revealed that FRA1 gene silencing decreased S phase-entry and delayed G2/M progression (Figure 1B). In agreement with the cell cycle defect, CyclinB1, a key G2/M cell cycle regulator, was significantly decreased in response to FRA1 loss, whereas CyclinD1, a G1-S phase regulator, was not changed (Figure 1C). The cell growth defect induced by FRA1 gene silencing was also observed in CAL27 and SCC25 HNSCC cells, as well as A431 epidermal SCC cells (Supplementary Figure 1). These results indicate that FRA1 has a key role in regulating HNSCC cell proliferation.

Soft agar colony formation is commonly used to assess anchorage-independent growth of cancer cells [33]. We found that FRA1 gene silencing significantly impaired soft agar colony formation of FaDu cells (Figure 1D). To further test whether FRA1 promotes cell survival, we performed an MTT assay to assess cell growth 3 days after incubation with varying doses of cisplatin, which is a chemotherapeutic agent frequently used for HNSCC treatment [1]. As expected, shFRA1 cells grew slower than control cells. In addition, these cells were more sensitive to cisplatin, as indicated by the reduced IC_50_ of 5.5 μM compared to 10 μM of control cells (Figure 1E). These results indicate that FRA1 plays a dominant role in HNSCC growth and resistance to chemotherapy.

### FRA1 promotes cell adhesion and migration

**Figure 2:**
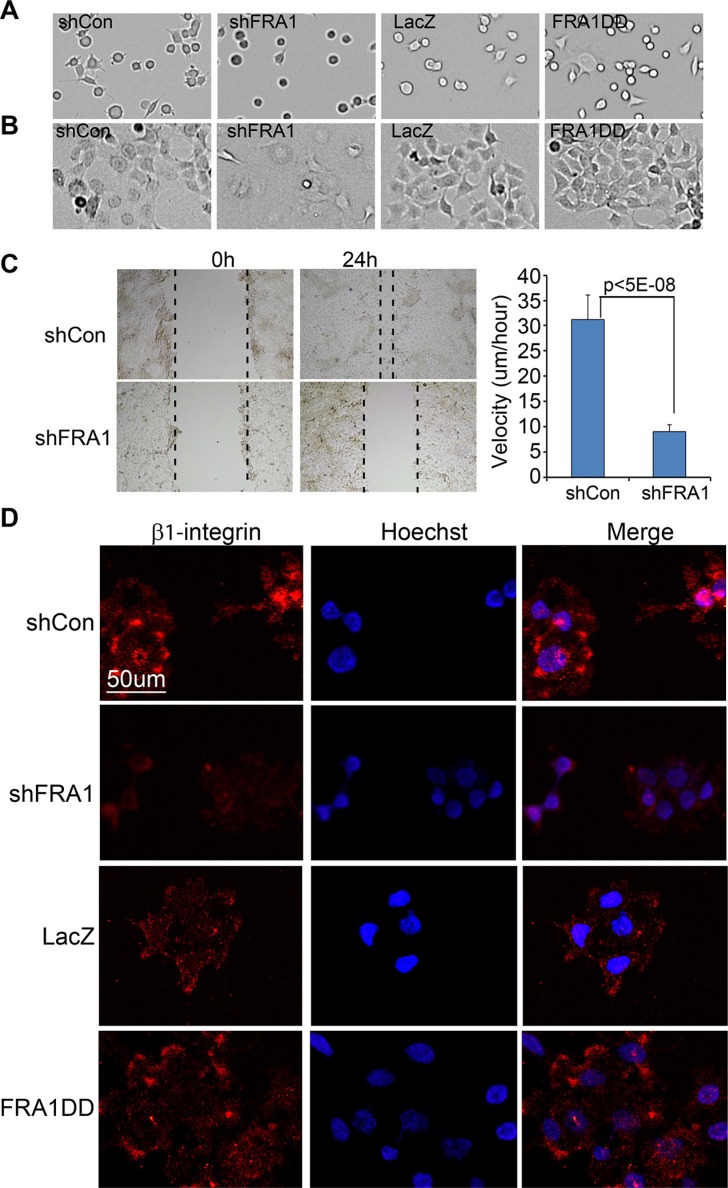
FRA1 promotes cancer cell adhesion and migration (**A**–**B**) Cell adhesion. FaDu cells transduced to express shCon, shFRA1, LacZ or FRA1DD were plated on 6-well dishes. Images of the attached cells were taken at (A) 1 h and (B) 24 h time-points. (**C**) Scratch-wound assay. FaDu cells transduced as above were grown to near confluence, and subject to 24 h serum starvation and then scratch-wounding. Images were taken under microscope at 0 h and 24 h time-points. Velocities were calculated under image J based on the real-time images taken between 10 h and 24 h time-points. Graph represents average velocity of 100 cells/condition + SD. (**D**) Immunostaining for cell surface β1-integrin. Fadu cells transduced to express shCon, ShFRA1, LacZ or FRA1DD were pre-incubated with serum free media at 37°C for 2 h and then incubated at 4°C with an antibody specific for active β1-integrin and subsequently with an Alexa 555-conjugated secondary antibody [orange] and counterstaining with Hoechst 33825 [blue].

Metastatic cancer cells often display increased rates of cell adhesion and migration. To determine whether FRA1 is involved in cell adhesion, we examined the rate of cell attachment and cell morphology at various time-points. At 1 h after seeding, less than 50% of shFRA1 cells compared to over 90% of control cells had attached to the dish, and the attached cells remained round (Figure 2A). Once fully attached at 24 h after seeding, shFRA1 cells appeared larger and less shiny under phase contrast microscope (Figure 2B), suggesting that FRA1 is important for cytoskeleton formation. Consistent with this idea, shFRA1 cells often lacked cell membrane protrusions that were commonly present in control cells as visualized by phalloidin staining of F-actin filaments (Supplementary Figure 2).

Next, we examined the effect of FRA1 on cell migration via a scratch wounding assay. FaDu cells were grown to near confluence, and subjected to cell growth arrest via serum starvation for 24 h and then scratch-wounding. We found that the wounds of the control cells were almost closed at 24 h after wounding, while those of shFRA1 cells were barely changed (Figure 2C). Quantitative microscopic real-time analysis of the wound edge cells showed that shFRA1 cells migrated with a significantly slower velocity (Figure 2C and Supplementary Video 1 and 2). Conversely, expression of the constitutively active FRA1 mutant, FRA1DD which contains point mutations of the phosphorylation sites (serine 252/265 to aspartate) [28], markedly accelerated cell migration (Supplementary Figure 3A). The FRA1-effects on cell migration were also readily observed in A431 cells (Supplementary Figure 3B). In order to characterize the migration defects at the molecular level, we performed live cell immunostaining with an antibody that specifically recognizes active β1-integrin, a key regulator of cell attachment and migration. As expected, the surface expression level of β1-integrin was markedly reduced in shFRA1 and increased in FRA1DD cells as compared to respective control cells (Figure 2D). These results indicate that FRA1 plays a positive role in cell attachment and migration.

### FRA1 promotes cell migration through JNK/c-Jun-dependent induction of KIND1

**Figure 3:**
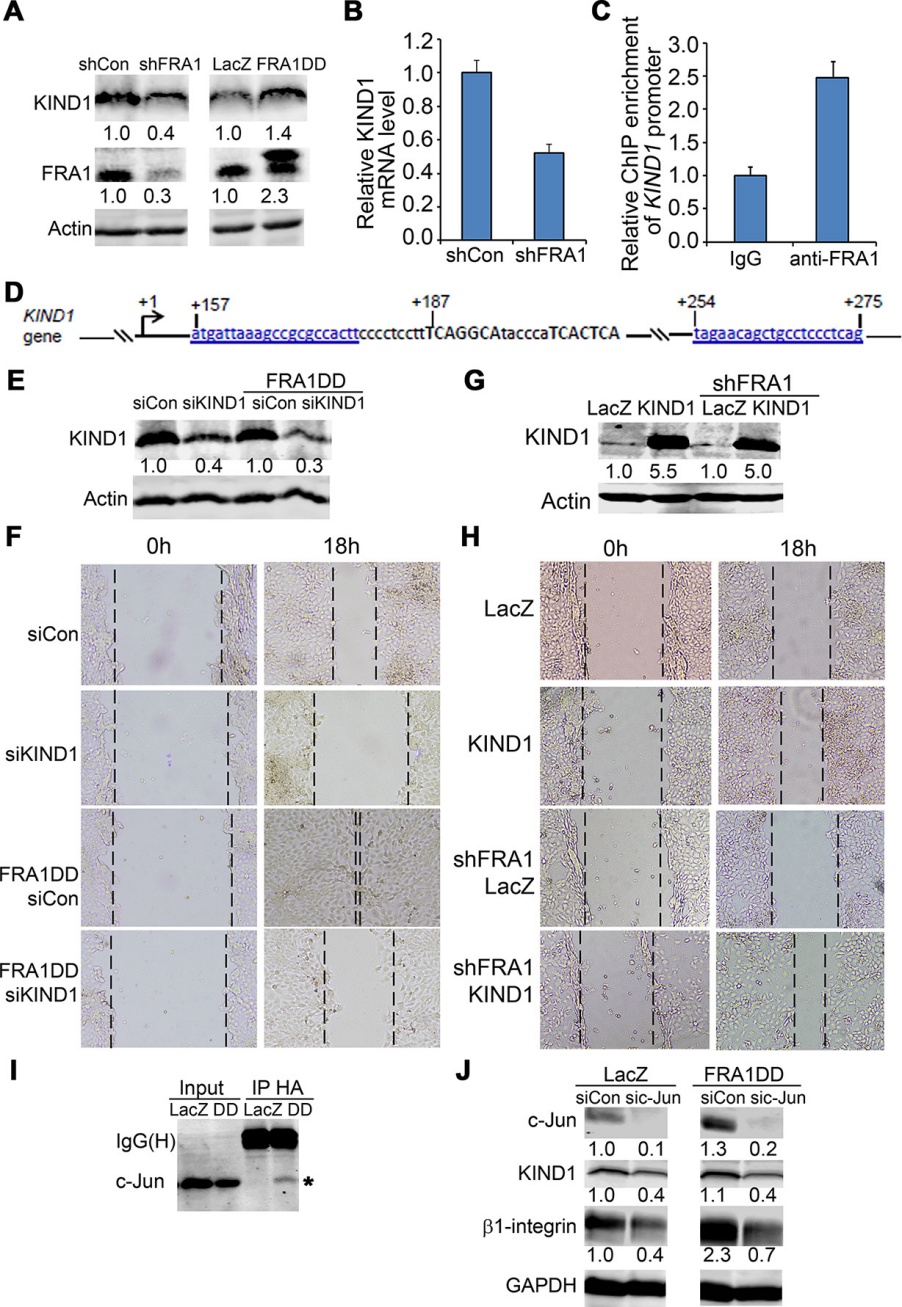
KIND1 is required for FRA1-promotion of cell migration (**A**) Immunoblotting of protein lysates isolated from FaDu cells transduced to express shCon, shFRA1, LacZ or FRA1DD. (**B**) KIND1 RT-PCR. Graph represents relative KIND1 mRNA levels + SD. (**C**) Diagram of *Kind1* gene (NCBI reference # NG_016213.1). Two putative AP-1 response elements shown in capital letters were located around 200 bp from *Kind1* gene transcription start site. (**D**) *Kind1* ChIP-PCR with an anti-FRA1 antibody and primers underlined and shown in blue above. Graph represents fold-enrichment by FRA1 antibody compared to control IgG + SD. (**E**) Confirmation of FRA1 gene silencing by immunoblotting. (**F**) Effect of KIND1 gene silencing on cell migration. Images were taken at 0 h and 18 h after scratch-wounding. (**G**) Confirmation of KIND1 expression by immunoblotting. (**H**) Effect of KIND1 overexpression on cell migration. **(I**) Co-immunoprecipitation (IP) of c-Jun with FRA1. Protein lysates were collected from FaDu cells expressing HA-tagged FRA1DD, and then subject to IP with an antibody against HA and then immunoblotting for c-Jun and FRA1. (**J**) Immunoblotting of protein lysates collected from FaDu cells expressing LacZ or FRA1DD together with siCon or sic-Jun. Relative densitometry shown below each band was obtained after normalization to that of respective loading control.

KIND1 is a cytoskeletal protein commonly expressed in epithelial cells, and is a key regulator for β1-integrin dynamics and turnover [34–36]. We asked whether KIND1 is involved in FRA1 regulation of cell migration. By immunoblotting, we found that KIND1 was decreased by FRA1 gene silencing, and increased by FRA1DD expression (Figure 3A). Quantitative RT-PCR confirmed a decrease of KIND1 mRNA in shFRA1 cells, indicating that KIND1 is induced by FRA1 at a transcriptional level (Figure 3B).

To determine whether KIND1 expression is directly controlled by FRA1 in an AP-1 dependent fashion, we performed chromatin immunoprecipitation (ChIP) with an antibody against FRA1 and then PCR with primers flanking two putative AP-1 response elements located about 200 bp from *Kind1* transcription start site (Figure 3C). We found that, as compared to control IgG, FRA1 antibody achieved a 2.5 fold enrichment of *Kind1* (Figure 3D), indicating that FRA1 physically interacts with the AP-1 cis-regulatory elements of *Kind1* gene.

Next, we examined the functional importance of KIND1. To do this, we first performed gene silencing of KIND1 using siRNA oligonucleotides in FaDu cells, as verified by immunoblotting (Figure 3E). Cell migration analysis showed that KIND1 gene silencing markedly slowed scratch wounding-induced cell migration of both control and FRA1DD expressing cells (Figure 3F). Conversely, overexpression of KIND1 enhanced control cell migration, and reduced the migratory defect caused by FRA1 loss (Figure 3G–3H). These results indicate that KIND1 is an important mediator of FRA1-promotion of cell migration.

Since FRA1 generally functions as heterodimers with Jun group AP-1 subunits, we asked whether c-Jun, a predominant Jun subunit, is required for FRA1-promotion of cell migration. By co-immunoprecipitation analysis, we found that FRA1DD indeed interacted with c-Jun (Figure 3I). Further immunoblotting showed that expression of FRA1DD increased KIND1 and β1-integrin, while gene silencing of c-Jun with siRNA oligonucleotides decreased their expression (Figure 3J). Consistently, c-Jun loss significantly slowed cell migration of both control cells and cells expressing FRA1DD (Supplementary Figure S4A). In agreement with the genetic data, pharmacological inhibition with the c-Jun upstream JNK inhibitor SP600125 abolished FRA1DD-promotion of cell migration (Supplementary Figure S4B). These results demonstrate that FRA1 partners with c-Jun to stimulate cell migration.

### FRA1 is required for AKT activation and increases CyclinB1 through ATK

**Figure 4:**
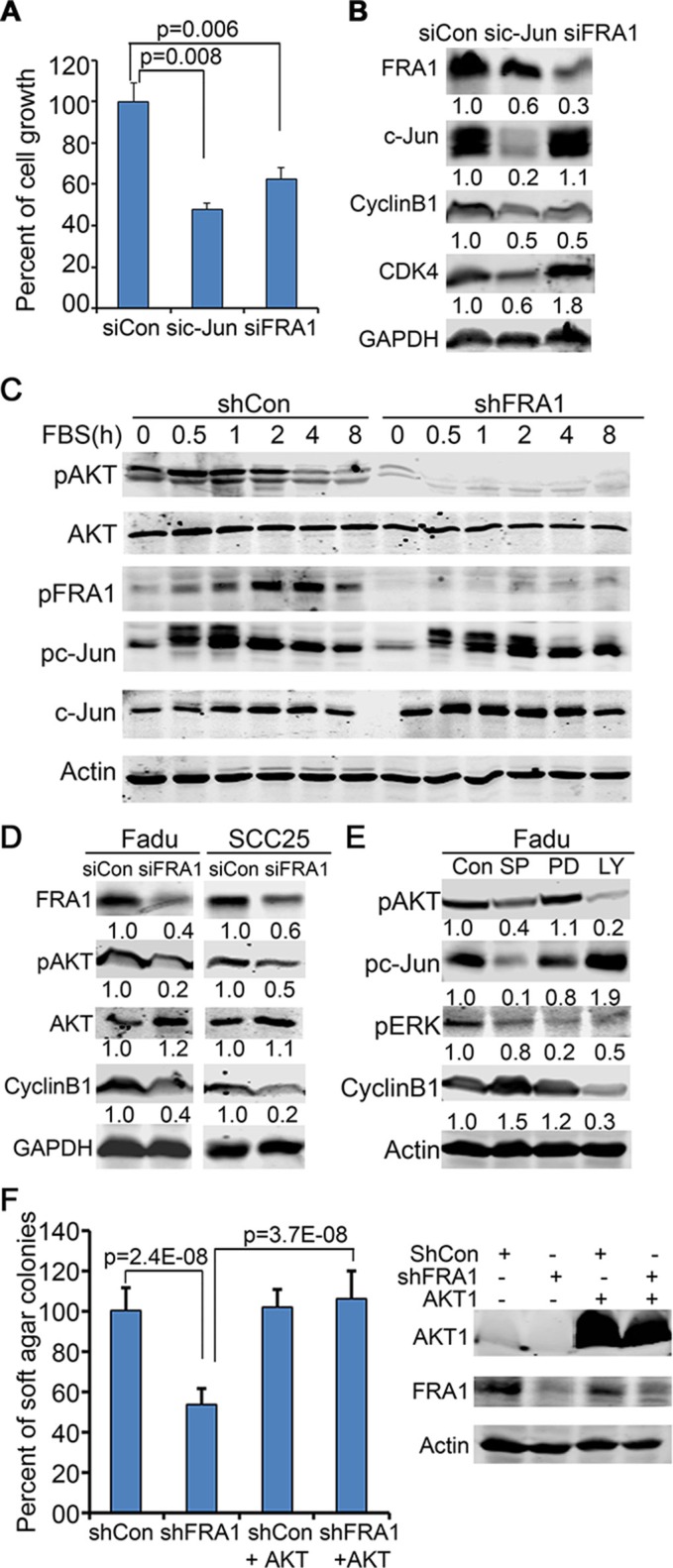
FRA1 is required for AKT-activation, and promotes cancer cell growth through AKT but not c-Jun (**A**) FaDu cells transfected with siCon, sic-Jun or siFRA1 oligonucleotides were seeded in triplicates for 48 h growth analysis. Graph represents average percentage of cell numbers normalized to control cells + SD. (**B–E**) Immunoblotting of protein lysates collected from (B) FaDu cells transfected as in (A), (C) FaDu cells that had undergone 48 h serum-starvation and then time-course stimulation with 25 ng/ml EGF, (D) FaDu and SCC25 cells transfected with siCon or siFRA1 oligonucleotides, and (E) FaDu cells that had been treated with JNK/c-Jun(SP600125), MEK/ERK(PD98059), PI3K/AKT(LY294002) inhibitors for 24 h. Relative densitometry was shown below each band. (**F**) Soft agar colony assay. FaDu cells transduced with lentiviruses for expression of shCon or shFRA1 either with or without the active form of AKT1. Graph represents average percentage of colonies normalized to shCon group + SD. Gene silencing and overexpression were confirmed by immunoblotting as shown on the right panel.

Both c-Jun and FRA1 have been found to have important roles in SCC [37]. Consistently, we found that c-Jun gene silencing also induced a cell growth defect in FaDu cells (Figure 4A). The question is whether FRA1-promotion of cell growth occurs in a c-Jun-dependent manner. By immunoblotting, we found that FRA1 loss decreased CyclinB1 but had a minimal effect on CDK4, whereas c-Jun loss decreased both CDK4 and CyclinB1, as well as FRA1 which is itself a target of c-Jun [38] (Figure 4B). To elucidate how FRA1 regulates CyclinB1, we examined the effects of FRA1 loss on EGF-induction of various signaling pathways in FaDu cells. To do this, cells were incubated with serum-free media for 48 h and then treated with EGF in a time-course manner. Immunoblotting revealed that pFRA1, pc-Jun and pAKT were apparently induced in control cells between 1 h and 8 h time-points after EGF-treatment. As expected, FRA1 and pFRA1 were reduced in shFRA1 cells. Surprisingly, pAKT induction was also markedly diminished in shFRA1 cells, while pc-Jun was induced normally (Figure 4C). In contrast, the total AKT levels remained stable between control and shFRA1 cells, indicating that AKT is regulated by FRA1 at the posttranscriptional level. The impaired AKT activation and CyclinB1 expression were also observed in FaDu and SCC25 cells transfected with siFRA1 oligonucleotides (Figure 4D), implicating that FRA1-regulation of AKT and CyclinB1 is likely a general mechanism in HNSCC cells.

AKT phosphorylation is required for CyclinB1 expression in prostate cancer cells [39]. To test whether AKT regulates CyclinB1 in HNSCC cells, we treated FaDu cells with a pharmacological AKT inhibitor LY294002 for 24 h, and then collected protein lysates for immunoblotting. We found that CyclinB1 was markedly decreased by LY294002 (Figure 4E). The reduced CyclinB1 expression in response to AKT-inhibition was also observed in A431, CAL27 and SCC25 cells (Supplementary Figure 5A, 5B). Overexpression of FRA1DD failed to prevent CyclinB1 downregulation by LY294002 (Supplementary Figure 5A), implicating that FRA1 acts upstream of AKT to regulate CyclinB1. In line with this notion, exogenous expression of a constitutively active form of AKT1 rescued the growth defect induced by FRA1 loss, as demonstrated by soft agar colony growth assay (Figure 4F). This result further confirms that FRA1 activates AKT to promote cancer cell growth.

### FRA1 is required for tumor growth and metastasis *in vivo*

**Figure 5:**
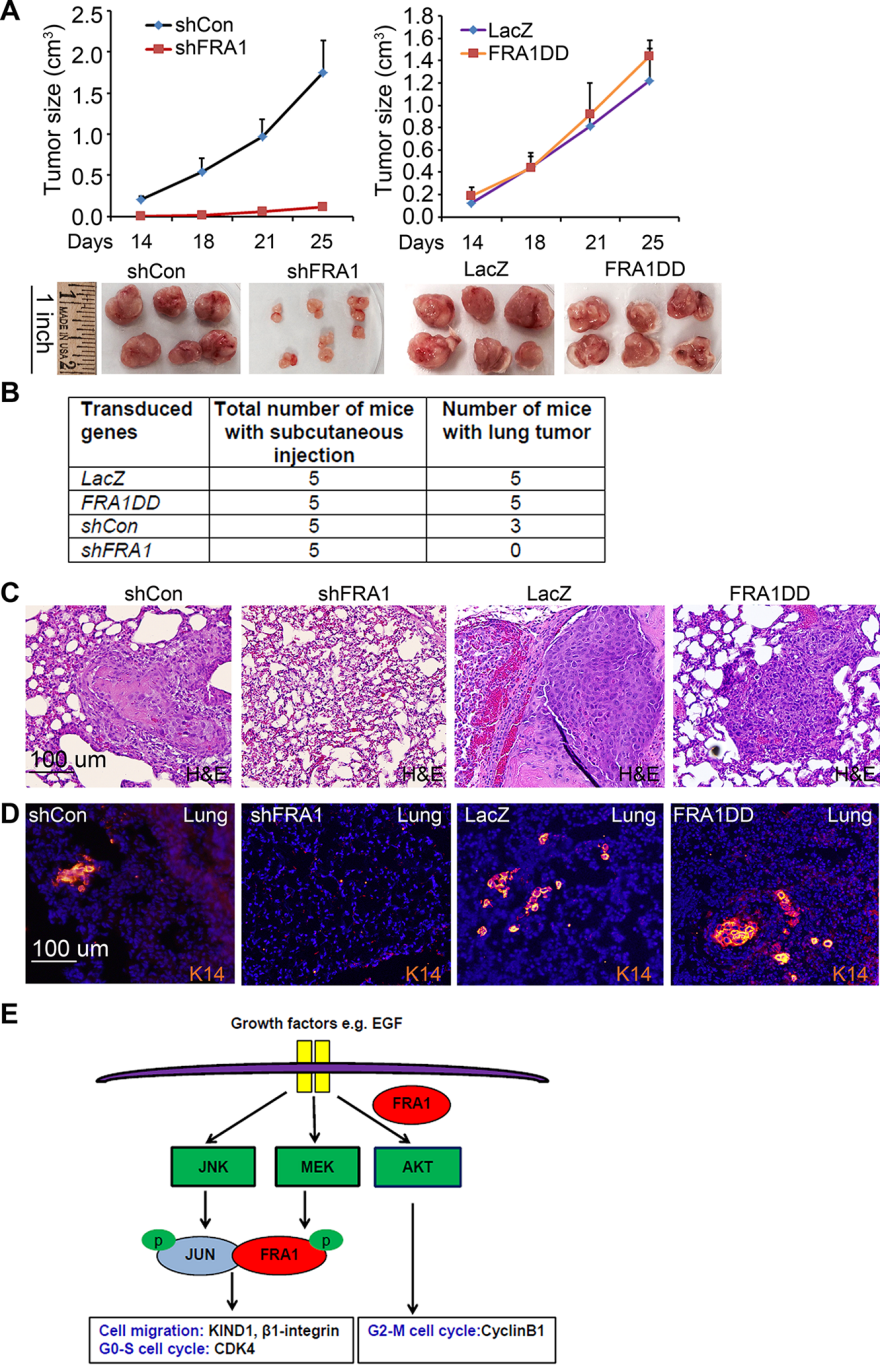
FRA1 is required for tumor growth and metastasis *in vivo* (**A**) Subcutaneous tumor growth. FaDu cells transduced to express shCon, shFRA1, LacZ or FRA1DD were subcutaneously injected to NSG mice. Graph represents average tumor volumes + SD. Tumors were photographed upon euthanasia. (**B**) Percent of animals with lung metastasis. (**C**) H&E staining of pulmonary tissues from animals that received subcutaneous injections as above. (**D**) Pulmonary tumors identified via immunostaining for K14 [orange], nuclei [Hoechst 33825]. (**E**) FRA1 working model depicting two distinct modes of FRA1-promotion of cell growth and migration.

To verify the relevance of these *in vitro* effects in an *in vivo* setting, we performed subcutaneous tumor growth analysis of FaDu cells in immunodeficient NSG mice. Consistent with the *in vitro* findings, shFRA1 cells displayed a significantly reduced tumor growth kinetic as compared to shCon cells (Figure 5A). Interestingly, cells expressing FRA1DD and LacZ control grew at similar rates (Figure 5A), indicating that endogenous FRA1 is sufficient for tumor growth. In agreement with the reduced growth phenotype, tumors of shFRA1 cells expressed significantly reduced levels of CyclinB1 and pAKT, as shown by immunofluorescent staining (Supplementary Figure 6).

To determine whether FRA1 promotes tumor metastasis, we collected and examined lung tissues from the euthanized animals 4 weeks after subcutaneous tumor inoculation. We found that 90% of the animals injected with control or FRA1DD-expressing cells had apparent tumor nodules in the pulmonary tissues as compared to 0% of those injected with shFRA1 cells (Figure 5B). These pulmonary tumors were identified by H&E staining as compact epithelia cell islands nested within the alveoli epithelium (Figure 5C). Immunostaining for the squamous epithelial cell marker, cytokeratin 14 (K14), further verified their SCC origin (Figure 5D). These results indicate that FRA1 plays a critical role in tumor growth and metastasis *in vivo*.

Taken together, these results reveal two distinct mechanisms of FRA1 regulation of tumor growth and metastasis (Figure 5E): 1) FRA1 acts through AKT to induce cell cycle regulators such as CyclinB1; and 2) FRA1 partners with the JNK effector c-Jun to induce expression of cell migration regulators including KIND1 and β1-integrin. Unlike c-Jun which is phosphorylated by JNK [40], FRA1 is phosphorylated in a MEK/ERK-dependent fashion [41, 42], which is a critical process in Ras-induced transformation of fibroblasts [43]. In agreement with these data, we showed that pFRA1 was reduced by MEK-inhibition with PD98059 (Supplementary Figure S7). Taken together, our findings identified FRA1 as a central integrator of MEK, JNK and AKT signaling pathways, pinpointing FRA1-induced signaling cascades as potential therapeutic targets.

## DISCUSSION

AP-1 family transcription factors are critical for a wide array of cellular processes, and display both distinct and overlapping functions among family members [44]. The Fos group subunits are unique in that they have to dimerize with the Jun-group proteins to be functional transcription factors. Compared to c-Fos, a well-characterized proto-oncogene, FRA1 does not have a distinct transactivation domain, which is thought to account for its inability to transform rodent fibroblasts [45]. Recently, it has become clear that FRA1 plays important roles in cancer [22]. It is overexpressed in many epithelial cancers, including cSCC (our unpublished data), HNSCC, thyroid, breast, lung, brain, esophageal, endometrial, prostate, colon, glioma and mesothelioma [22, 30, 46]. The functional importance of FRA1 has been documented in several of these cancers in the areas of cell proliferation, migration and survival, as well as tumor microenvironment. Nevertheless, most of the molecular mechanisms characterized so far are linked to AP-1 function. It is intriguing to find that FRA1 regulates CyclinB1 indirectly through AKT and in a c-Jun-independent manner. Presumably, AKT regulates CyclinB1 through transcription factors other than c-Jun and FRA1. It is worth noting that, in contrast to AKT, JNK inhibition with SP600125, resulted in a slight increase of CyclinB1, though the specificity and importance of such an effect are yet to be examined.

The PI3K/AKT signaling pathway is mutated in over 30% of HNSCC tumors, and plays a critical role in HNSCC growth and progression [15, 16]. This work identified a novel mechanism that presumably contributes to sustained AKT activation in HNSCC tumors that have no clear genetic mutations in the PI3K pathway [15]. It is likely that a similar molecular paradigm contributes to cSCC which shares many similarities with HNSCC in pathogenesis [17]. Our findings are also in line with earlier studies linking cytoplasmic c-Fos and FRA1 to the synthesis of membrane lipid polyphosphoinositides including PIP1 and PIP2 [31, 32], components essential for PI3K activation. In agreement with a role of FRA1 in membrane lipid synthesis, FRA1 was located on the cell membrane of tumor marginal cells (Data not shown). It has yet to be determined whether c-Fos and FRA1 play non-redundant roles in regulating PIP1 and PIP2 synthesis, and whether FRA1 binds lipid synthesis enzymes such as CDP-diacylglycerol synthase and phosphatidylinositol (PtdIns) 4-kinase II alpha, as c-Fos does [47].

CyclinB1 is induced in a cell cycle-dependent manner [48], and is suppressed by p53 [49]. Future studies may be directed to elucidating whether p53 or another gene regulator is responsible for AKT-effect on CyclinB1 expression. Of further interest, FRA1 has been characterized as an AKT-inducible gene in prostate cancer cells and also an AKT-downstream effector responsible for development of trophoblast lineages [50, 51]. Nevertheless, we found that overexpression of FRA1DD failed to rescue CyclinB1 expression in lieu of PI3K/AKT inhibition, indicating that FRA1 acts upstream of AKT in HNSCC cells.

The β1-integrin signaling pathway is important for cell migration and metastasis. In agreement with this, β1-integrin signaling was reduced in response to FRA1 loss. Additionally, this work identified KIND1 as a new transcriptional target gene of FRA1 and c-Jun, and as an important mediator of FRA1 regulation of β1-integrin signaling and cell migration. Nevertheless, we note that FRA1-promotion of cell migration likely involves other AP-1 target genes such as MMPs and adenosine receptor A2B (ADORA2B) as described in breast cancer [52, 53], uPA in colon carcinoma [54], receptor tyrosine kinase AXL in bladder cancer [28], and CD44 and c-Met in mesothelioma [55]. It is plausible that some of these mechanisms are relevant to HNSCC.

The Ras/MEK signaling pathway is almost ubiquitously activated in cancer cells. Thus, MEK-dependent phosphorylation of FRA1 is likely relevant to many cancers beyond HNSCC. In addition, FRA1 is transcriptionally induced by a number of cancer relevant transcription factors including c-Jun, c-Fos and c-Myc [22], and epigenetic modulators such as histone demethylase KDM4A [37]. It is highly conceivable that FRA1 acts through AP-1-dependent and independent mechanisms to regulate a wide array of molecules involved in cell proliferation, migration, mesenchymal transdifferentiation and cancer cell plasticity of many other cancers [26, 56]. Besides cancer cell autonomous effects, FRA1 may also play a role in the tumor microenvironment by acting in endothelial cells to enhance vascularization and in immune cells to indirectly mediate cancer cell motility and invasion [24, 57, 58].

FaDu cells with FRA1 gene silencing were more sensitive to cisplatin than control cells, which suggest that FRA1 has a protective role during stress responses, and may therefore be targeted to enhance chemotherapy. These results are in agreement with the data showing that FRA1 is essential for the survival of Ras-transformed thyroid cancer cells [59]. In contrast, FRA1 is not critical for animal survival and growth [25], which suggests that FRA1-targeted therapies may not induce major side effects on normal cell growth and survival.

In summary, this work established that FRA1 is a central integrator of the JNK, MEK and AKT signaling pathways, and functions as a critical oncogene and biomarker in cSCC and HNSCC. Given the broad relevance of FRA1 to the multitude of epithelial cancers, findings of this study provide general insights to diagnostic and therapeutic developments for many other cancers.

## MATERIALS AND METHODS

### Cell culture and gene transfer

FaDu, SCC25, CAL27 and A431 cells were obtained from (ATCC, Manassas, VA), and maintained at a 37°C incubator supplemented with 5% CO2. FaDu cells were cultured in Eagle's Minimum Essential Medium with 10% fetal bovine serum, and SCC25, CAL27 and A431 cells were cultured in Dulbecco's Modified Eagle Medium with 10% fetal bovine serum (Life Technologies, Grand Island, NY). The epithelial origin of these cells was verified by immunostaining for cytokeratin 14 (K14). The retroviral LZRS-FRA1DD construct was generated by insertion of the BamH I and Not I fragment of the DNA encoding FRA1DD into the LZRS vector [28]. The LZRS-KIND1 plasmid was generated as described [35]. For stable gene expression or silencing, cells were transfected with the expression constructs encoding FRA1DD, KIND1 or LacZ control using the GenJet Plus reagent (SignaGen Laboratories, Ijamsville, MD), or transduced with the shRNA lenti-virus targeting FRA1 (clone TRCN0000019541, Duke RNAi Screening Facility, Durham, NC) or the non-silencing control, and then selected with 1 μg/ml puromycin for 1–2 weeks. For AKT expression, cells were infected with the lentivirus encoding a constitutively active AKT1 mutant as described [60]. For transient gene silencing, siRNA oligonucleotides targeting FRA1 (Cat# S15583), KIND1 (Cat# HSS124666) or c-Jun (Cat# S16960) were obtained from (ThermoFisher Scientific, Waltham, MA).

### Cell growth, adhesion, scratch-wounding and MTT assays

For cell growth analysis, transfected or infected FaDu cells were plated in triplicates on 12-well dishes (5 × 10^4^/well), and trypsinized for cell counting 3 days later. For the adhesion assay, images of the attached cells were taken at 1 h or 24 h post-seeding. For the scratch-wound assay, cells were grown to near confluence, incubated with serum free media overnight, and then wounded with a 10-μl pipette tip. Images were taken at 0 and 18 h or 24 h post-wounding. For migration velocity analysis, live images were taken between 10 h and 24 h after wounding at 5 minute intervals on a Leica microscope with temperature, CO2 and humidity controls. Velocity was calculated using the NIH Image J program. For cisplatin response analysis, cells were seeded on 96-well dishes at 20,000 cells/well, treated in hexad with 0, 5, 10, 20 and 50 μM cisplatin (Sigma-Aldrich, St. Louis, MO) for 72 h, and then incubated with 10 μl/well of 5 mg/ml MTT [3-(4,5-Dimethylthiazol-2-yl)-2,5-diphenyltetrazolium bromide] for 2 h, after which the medium was replaced with 200 μl of DMSO. The absorbance was read at 570 nM in the BioRad plate reader.

### Soft agar colony formation

Soft agar colony formation was performed in a similar manner as described [61]. Briefly, FaDu cells transduced to express shCon and shFRA1, together with or without active AKT1, were plated in triplicates with 0.35% Nobel agar at 50,000 cells/well in 6-well dishes that were preloaded with 2 ml 0.5% Nobel agar/well. Colonies were visualized by crystal violet staining 2 weeks later.

### Flow cytometry analysis

Transduced FaDu cells were treated with 100 μg/ml BrdU for 1.5 h, and then trypsinized and transferred to 96-well v-bottom plates for fixation with formaldehyde and permeabilization with 0.1% Triton X-100/0.1 M HCl on ice. After a brief wash with 0.15 mM NaCl and 15 mM trisodium citrate dehydrate, cells were heated to 95°C for 5 minutes to denature DNA, incubated with the AF-647 conjugated rabbit anti-BrdU antibody (Invitrogen) for 1 h, and then treated with propidium iodide (25 μg/ml) and RNase A (100 ng/ml) for 30 minutes. After incubation, cells were washed and resuspended in 100 μl PBS for flow cytometry analysis using BD Canto II flow cytometer. Cell cycle studies were performed with 3 biological replicates, each with 3 technical replicates.

### Animal studies

Animal studies were performed in accordance with protocols approved by Duke Animal Care and Use Committee. Immunodeficient NSG mice were purchased from Duke Cancer Center Isolation facility (Durham, NC). For subcutaneous injection, FaDu cells were suspended at 1 × 10^5^ cells/200 μl PBS:matrigel (3:1) for each injection. Tumors were measured biweekly for 3 weeks, and collected from euthanized animals at the end-point.

### Immunoblotting

Protein lysates (20 μg/sample) prepared either with RIPA buffer or SDS-PAGE loading buffer were used for immunoblotting with antibodies against FRA1 and actin (Santa CruZ Biotechnology, Santa CruZ, CA), pFRA1, pc-Jun(S73), c-Jun(Ab-243), pERK, pFAK and β1-integrin (Cell Signaling Technology, Danvers, MA), pAKT(Th308) (Genscript, Piscataway, NJ), KIND1 (Sigma) and AKT(Ab-308) (Abcam, Cambridge, MA). The blots were detected with IRDye-conjugated secondary antibodies (Invitrogen), and imaged with the Odyssey Imagining system (Li-COR, Lincoln, NE).

### ChIP-PCR and RT-PCR

ChIP-PCR was performed as described [62]. Briefly, genomic DNA isolated from FaDu cells were used for immunoprecipitation with an antibody against FRA1 or control IgG (Santa Cruz Biotechnology), and then used for PCR with primers designed to amplify the AP-1 consensus element located on *Kind1* promoter (Forward 5′-atgattaaagccgcgccactt-3′ and reverse 5′-ctgagggaggca gctgttcta-3′). For RT-PCR, total RNA was isolated from FaDu cells transduced to express shCon or shFRA1, and then used for RT-PCR with *Kind1* cDNA primers (Forward 5′-gatgggtatgcctgaaagga and reverse 5′-tcttgcgatgtctcatcctg-3′) and GAPDH as internal controls as described [62].

### Histology and immunostaining

Hematoxylin and eosin (H&E) staining was performed with paraffin sections by the Duke Pathology lab (Durham, NC). Immunofluorescent staining was performed with frozen tissue sections or cells grown on glass cover slips as previously described (Zhang et al., 2007; Ke et al., 2010). Tissue sections were fixed with cold methanol, and cultured cells were fixed with 10% buffered formalin/4% formaldehyde followed by permeabilization with 0.1% triton-X 100. The primary antibodies against pFAK, CyclinB1, pAKT and IgG control were from (Cell Signaling Technology), and Alex- 555 or 488-conjugated secondary antibodies were obtained from (Fisher Scientific, Grand Island, NY). Samples were counterstained with Hoechst 33825 (Sigma), and imaged under the Olympus BX41 microscopic imaging system (Center Valley, PA). For detection of active β1-integrin in live cells, FaDu cells transduced to express shCon, ShFRA1, LacZ or FRA1DD were preincubated with serum free media at 37°C, washed two more times with the same media, and then incubated at 4°C with an antibody that recognize active β1-integrin (EMD Millipore, Billerica, MA) followed by detection with an Alexa 555-conjuated secondary antibody and counterstaining with Hoechst 33825.

### Statistical analysis

Data represented the average values ± a standard deviation of at least 3 separate experiments. *P*-values were obtained using Student *T*-test. A *p*-value of < 0.05 is considered statistically significant.

## SUPPLEMENTARY MATERIALS FIGURES AND TABLES






