# Supplementary material for: FRA1 promotes squamous cell carcinoma growth and metastasis through distinct AKT and c-Jun dependent mechanisms
Source: Oncotarget. 2016 Apr 29;7(23):34371–83. doi: 10.18632/oncotarget.9110 (PMC5085162; doi:10.18632/oncotarget.9110)
Supplement: Supplementary file 1 [file oncotarget-07-34371-s001.pdf]

FRA1 promotes squamous cell carcinoma growth and metastasis through distinct AKT and c-Jun dependent mechanisms

Supplementary Materials

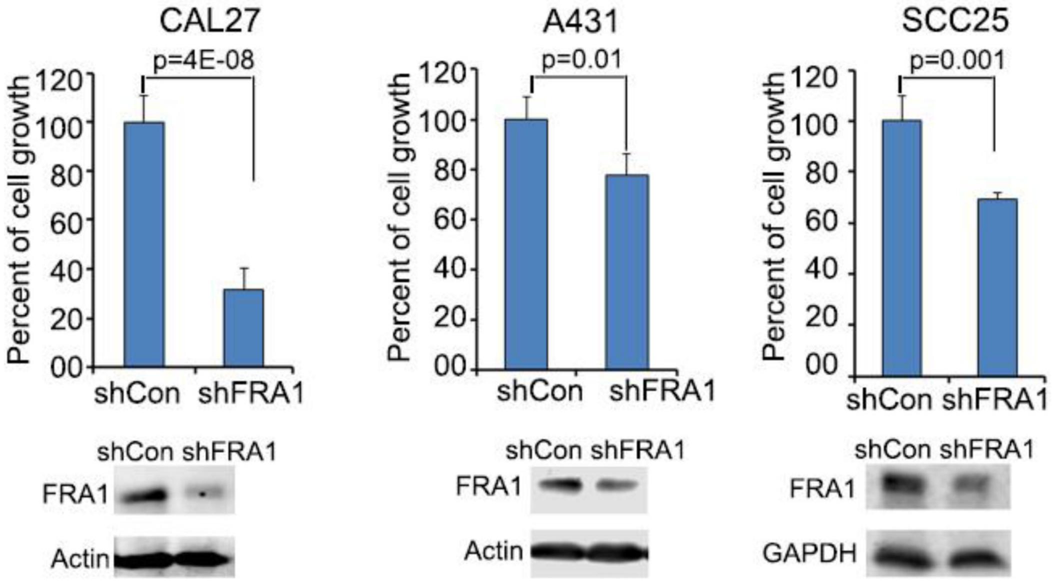

Supplementary Figure S1: Gene silencing of FRA1 inhibits SCC cell growth. CAL27 and A431 cells were transduced for expression of shRNA targeting FRA1 (shFRA1) or nonsilencing control (shCon). Cells were then seeded in triplicates for 48 h growth analysis. Graph represents average percentage of cell numbers normalized to control cells + SD. Gene silencing was confirmed by immunoblotting shown below each graph.

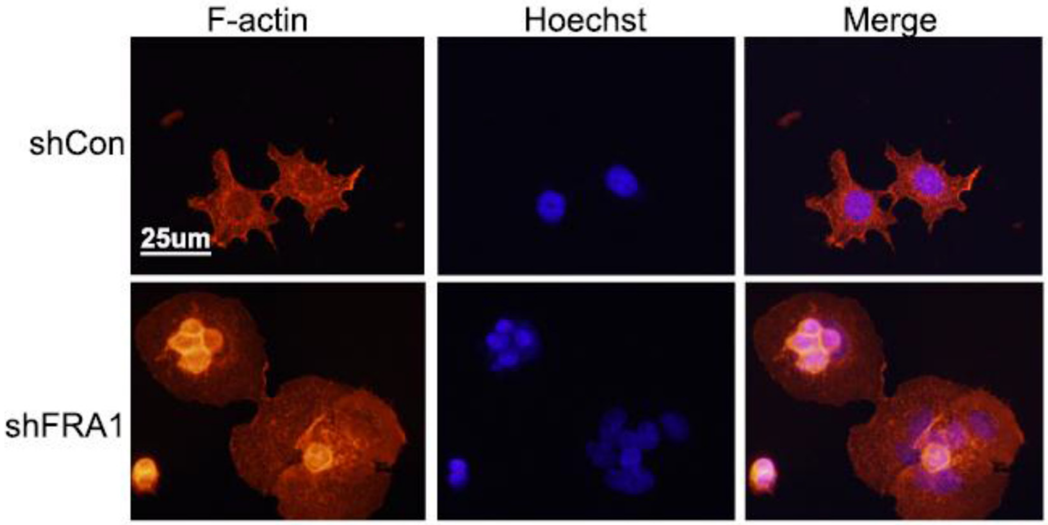

Supplementary Figure S2: FRA1 enhances cytoskeleton assembly. TRITC-conjugated Phalloidin staining of F-actin fiber in Fadu cells expressing shCon or shFRA1. F-actin [orange], nuclei [Hoechst 33825].

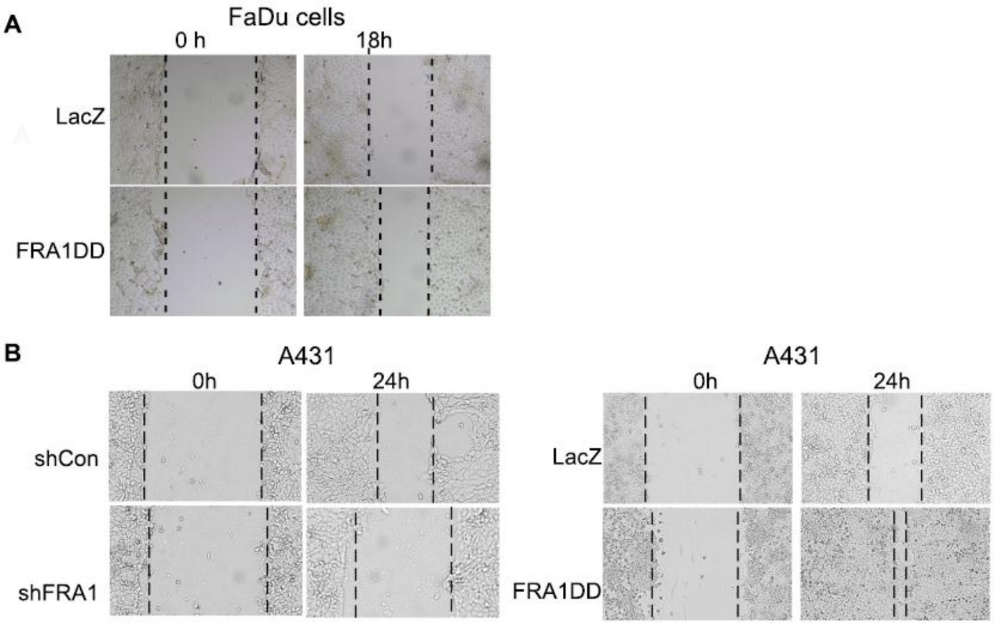

Supplementary Figure S3: FRA1 promotes SCC cell migration. (**A**–**B**) Scratch-wounding induced cell migration. (**A**) FaDu cells transduced to express LacZ or FRA1DD or (**B**) A431 cells transduced to express shCon, shFRA1, LacZ or FRA1DD, or transfected with siCon or siFRA1 oligonucleotides were grown to near confluence, and subject to 24 h serum-starvation and then scratch-wounding. Images were taken under microscope at indicated time-points.

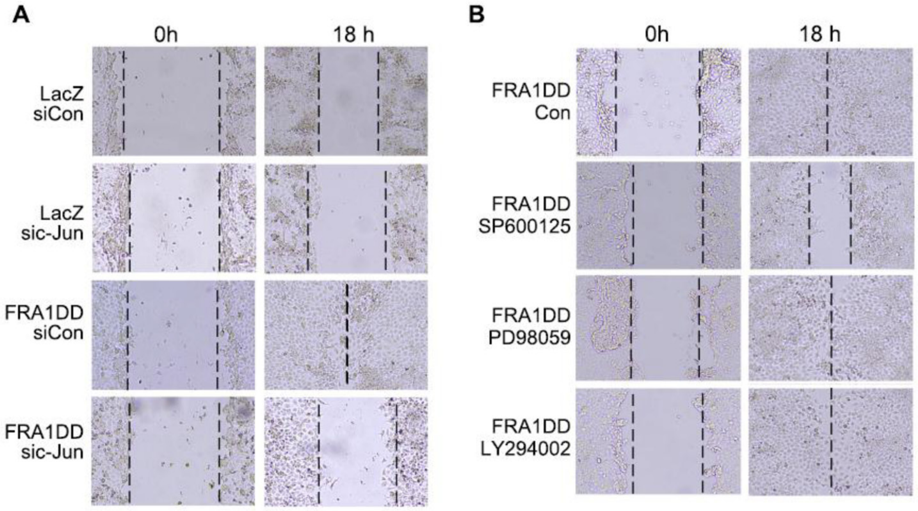

Supplementary Figure S4: FRA1 stimulates cell migration in a JNK/c-Jun-dependent manner. (**A**). Scratch-wounding assay. FaDu cells transduced to express FRA1DD were transfected with siCon and sic-Jun oligonucleotides, and then set up for scratch-wounding assay. (**B**) Scratch-wounding assay. FaDu cells expressing FRA1DD were set up for scratch-wounding assay as in (b), and then treated with SP600125 (10 μM), PD98059 (20 μM) and LY2940002 (25 μM) after wounding. Images were taken under microscope at 0 h and 18 h time-points.

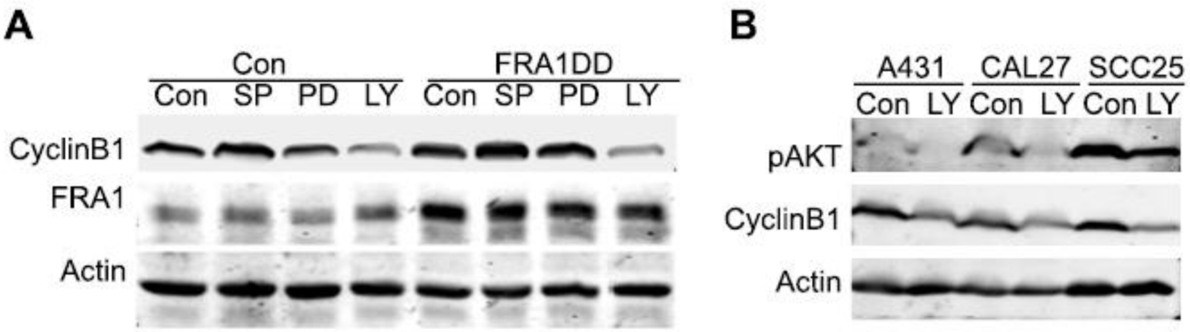

Supplementary Figure S5: AKT activation is required for CyclinB1 expression in in SCC cells. (**A**) Immunoblotting of protein lysates collected from control or FRA1DD expressing cells that had been treated with SP600125 (10 μM), PD98059 (20 μM) and LY2940002 (25 μM) Fadu cells for 24 h. **(B)** Immunoblotting of protein lysates collected from A431, CAL27 and SCC25 cells that had been treated with 25 μM PI3K/AKT inhibitor (LY294002) for 24 h.

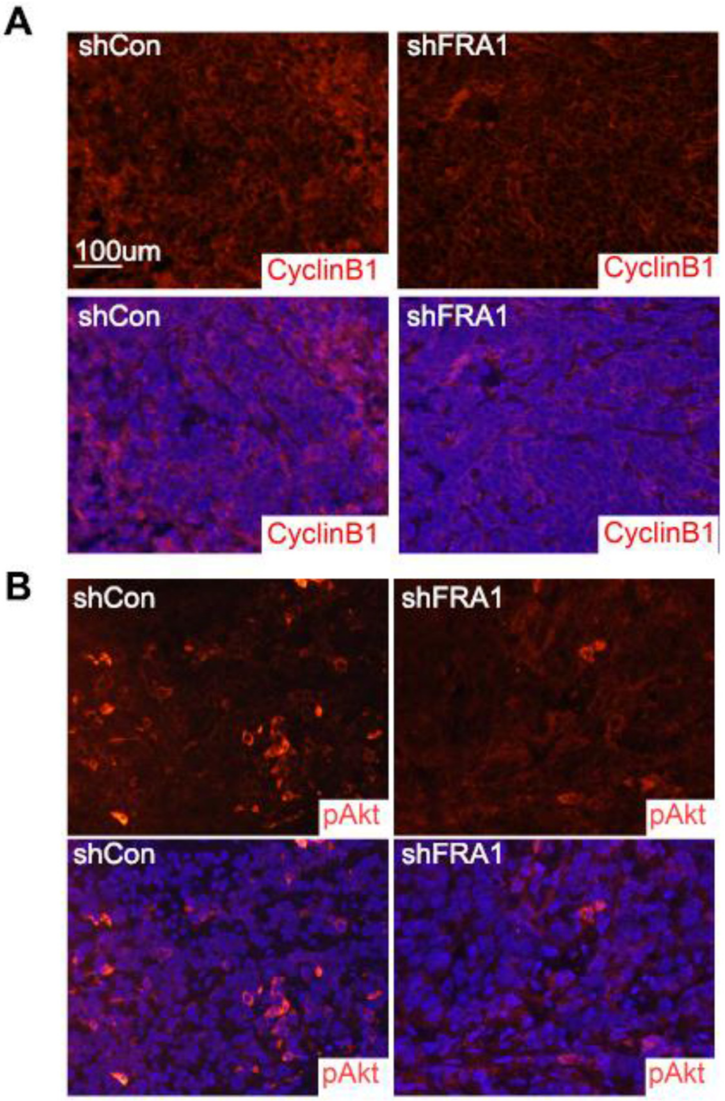

Supplementary Figure S6: FRA1 gene silencing decreases CyclinB1 and pAKT expression in subcutaneous tumors. Frozen tissue sections of subcutaneous tumors expressing shCon or shFRA1 were immunostained with primary antibodies against CyclinB1 and pAKT, and detected with Alexa 555-conjugated secondary antibodies [orange]. Nuclei [blue, Hoechst 33825].

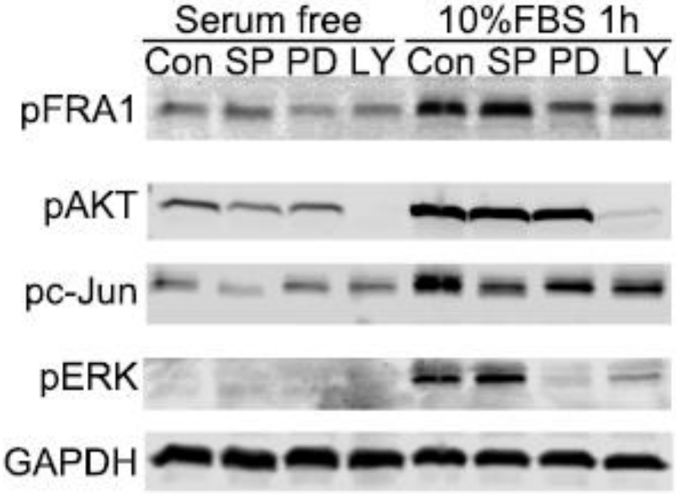

Supplementary Figure S7: FRA1 is subject to MEK-dependent phosphorylation. Immunoblotting for pFRA1, pAKT, pc-Jun, pERK and Actin of protein extracts isolated from FaDu cells treated with DMSO control (Con) or pharmacological inhibitors targeting JNK (SP600125), MEK (PD98059), or PI3K (LY294002).

Supplementary Video S1: FRA1 is required for FaDu cell migration. FaDu cells transduced to express shCon were cultured to near confluence and serum starved for 24 h. Cells were then scratch-wounded and subject to real-time live cell imaging under microscope for between 10 and 24 h after wounding.

Supplementary Video S2: FRA1 is required for FaDu cell migration. FaDu cells transduced to express shFRA1 were cultured to near confluence and serum starved for 24 h. Cells were then scratch-wounded and subject to real-time live cell imaging under microscope for between 10 and 24 h after wounding.
